# Potential Use of Autologous Renal Cells from Diseased Kidneys for the Treatment of Renal Failure

**DOI:** 10.1371/journal.pone.0164997

**Published:** 2016-10-24

**Authors:** Sunil K. George, Mehran Abolbashari, John D. Jackson, Tamer Aboushwareb, Anthony Atala, James J. Yoo

**Affiliations:** 1 Wake Forest Institute for Regenerative Medicine, Wake Forest School of Medicine, Medical Center Boulevard, Winston-Salem, North Carolina, 27157, United States of America; 2 Texas Tech University, Paul L. Foster School of Medicine, El Paso, Texas, 79905, United States of America; 3 RSS Urology—Mid Atlantic, Allergan Medical Affairs, P.O. Box 19534, Irvine, California, 92623, United States of America; National Cancer Institute, UNITED STATES

## Abstract

Chronic kidney disease (CKD) occurs when certain conditions cause the kidneys to gradually lose function. For patients with CKD, renal transplantation is the only treatment option that restores kidney function. In this study, we evaluated primary renal cells obtained from diseased kidneys to determine whether their normal phenotypic and functional characteristics are retained, and could be used for cell therapy. Primary renal cells isolated from both normal kidneys (NK) and diseased kidneys (CKD) showed similar phenotypic characteristics and growth kinetics. The expression levels of renal tubular cell markers, Aquaporin-1 and E-Cadherin, and podocyte-specific markers, WT-1 and Nephrin, were similar in both NK and CKD kidney derived cells. Using fluorescence- activated cell sorting (FACS), specific renal cell populations were identified and included proximal tubular cells (83.1% from NK and 80.3% from CKD kidneys); distal tubular cells (11.03% from NK and 10.9% from CKD kidneys); and podocytes (1.91% from NK and 1.78% from CKD kidneys). Ultra-structural analysis using scanning electron microscopy (SEM) revealed microvilli on the apical surface of cultured cells from NK and CKD samples. Moreover, transmission electron microscopy (TEM) analysis showed a similar organization of tight junctions, desmosomes, and other intracellular structures. The Na^+^ uptake characteristics of NK and CKD derived renal cells were also similar (24.4 mmol/L and 25 mmol/L, respectively) and no significant differences were observed in the protein uptake and transport characteristics of these two cell isolates. These results show that primary renal cells derived from diseased kidneys such as CKD have similar structural and functional characteristics to their counterparts from a normal healthy kidney (NK) when grown *in vitro*. This study suggests that cells derived from diseased kidney may be used as an autologous cell source for renal cell therapy, particularly in patients with CKD or end-stage renal disease (ESRD).

## Introduction

Chronic kidney disease (CKD) is a global health problem, which can lead to end-stage renal failure and eventually death if not treated [1, 2]. Approximately 8–16% of the adult population suffers from CKD which results in reduced glomerular filtration rate, increased urinary albumin excretion, interstitial fibrosis, anemia and hyperphosphatemia [3, 4]. Patients with CKD frequently have additional complications, such as cardiovascular disease, type-2 diabetes, and/or hypertension [5, 6]. According to the U.S. Centers for Disease Control and Prevention (CDCP), in 2011, over 100,000 patients began treatment for End-Stage Renal Disease (ESRD); furthermore, diabetes or hypertension were identified as the leading cause for 70% of new cases of ESRD [3].

Currently, the standard treatment for renal failure is either kidney transplantation or dialysis. However, problems associated with transplantation, which include donor organ shortage, rejection and life-long immunosuppression, remain a challenge. In addition, immunosuppression can result in increased infections and potential tumor formation. Dialysis replicates the renal filtering process by removing toxic substances from the blood. Unfortunately, this can result in anemia, low blood pressure, increased potential for infection and failure to restore other necessary renal functions, such as erythropoietin production and activation of vitamin D [7]. It also contributes to the overall inefficient recovery of renal function and morbidity. All these point to an urgent need to develop alternative strategies for the treatment of CKD.

The field of regenerative medicine offers hope with novel combinations and standalone approaches to the structural and functional repair and regeneration of diseased organs using various cells, scaffolds, and biological factors [8–10]. Specifically, cell-based therapies have been proposed to treat a variety of human diseases and medical conditions: transplantation of pancreatic islet cells in diabetes patients [11]; autologous chondrocytes for cartilage repair [12]; B cell therapy for systematic lupus erythematosus [10, 13]; hematopoietic stem cell transplantation for cancer treatment [14]; and stem/progenitor cell transplant for neuronal disease [15].

The kidney consists of more than 20 different cell types that are structurally organized into distinct anatomical and functional compartments [16], including the main components of the nephron which are the proximal and distal tubules. Proximal tubular cells (PTCs) are the dominant cell type found in the kidney, and play important roles in reabsorption of proteins and electrolytes, hydrolase activity and erythropoietin (EPO) production in interstitial fibroblast-like cells in the cortex and outer medulla of the kidney [17–19] We previously studied that erythropoietin (EPO)-producing primary human renal cells showed renoprotective properties. In addition, we also observed that human renal cells reintroduced in an artificial renal device resulted in the formation of renal structures and produced urine-like fluid [8, 20]. Thus, it has been postulated that replacing or recovering renal cells can promote general renal recovery. Studies have demonstrated that renal cells containing PTCs exhibit high proliferative capacity and can promote renal regeneration in rodent models of acute renal failure (ARF) [21, 22]. In addition, adult renal stem cells and progenitor cells have been derived from the renal papilla and Bowman’s capsule of adult human kidneys and ameliorate the structural recovery of the kidney after the induction of ARF, [23–25]. The development of a renal epithelial cell therapy would be of significant value given the current suboptimal treatments available for renal failure. Given all that is known about the importance of PTCs in the kidney and the initial success of renal cell therapy treatments in AKF models, we believe that renal cells containing PTCs could be used to treat CKD. To this end, we have established a cell culture method that enables the expansion of primary renal cells from human tissues. Using this process, we have determined that the majority of the cell population consisted of PTCs and to a lesser degree podocytes [9, 26]. This population of primary CKD cells include multiple cell types that could proliferate *in vitro* similar to NK cells; use of these cells for treatment of CKD could potentially lead to functional recovery of the renal tissue due to integration of these cells into sites of injury in the CKD kidney.

Although human renal cell therapies are still in experimental stages they seem to have great potential. Autologous cell therapies that target the innate ability of renal cells for repair and regeneration, either via paracrine effects or environmental modification, could provide a more effective alternative approach to currently available therapies. Immunogenicity, teratogenicity, and ethical concerns that are associated with the use of stem cells, particularly embryonic stem cells, could be avoided by using an autologous cell source. As a result, the aim of the present study was to investigate whether primary renal cells isolated from diseased kidneys (CKD) are physiologically similar to primary cells isolated from normal kidneys (NK). In such case, renal cells from a diseased kidney could be used as an autologous cell source for renal cell therapy in CKD and ESRD patients.

## Materials and Methods

### Human Renal Cell Culture

Donor human kidneys not used for transplantation were obtained from Carolina Donor Services (Winston-Salem, NC, USA), with written consent from the donors and ethical approval by the Institutional Review Board of Wake Forest University Health Sciences. Three normal kidneys (NK) and three kidneys from donors with CKD were used (Table 1). The medullary region of the kidney was removed and the cortical tissue cells were isolated. [9–10] Briefly, the kidney (cortex) was placed in Krebs-Ringer bicarbonate buffer (Sigma, St. Louis, MO, USA) supplemented with 1% antibiotic (penicillin-streptomycin, Gibco Invitrogen, Carlsbad, CA, USA). Renal capsules and adjacent connective tissues were removed using scissors to prevent contamination of unwanted cell types. The remaining tissue was minced and enzymatically digested using Liberase Blendzyme (Roche, Indianapolis, IN, USA) for one hour at 37°C in a shaking water bath. The suspension was then filtered using a 100μm cell strainer (BD Falcon, San Jose, CA, USA) and centrifuged at 1500 rpm for 5 minutes. The cell pellet was re-suspended in culture media (1:1 mixture of keratinocyte serum-free medium (KSFM) and premixed Dulbecco’s Modified Eagle’s Medium (DMEM), supplemented with 5% fetal bovine serum (FBS), 1% penicillin-streptomycin, 1% glutamine (100x), 0.4% insulin transferrin selenium (ITS), 0.25% EGF, and 0.25% bovine pituitary extract) and plated in a 15 cm^2^ cell culture plate. The cells were incubated at 37°C with 5% CO_2_, and the medium was changed every three days. The cells were sub-cultured for expansion at a ratio of 1:3 when confluent.

**Table 1:** Summary of donor information and disease status.

Kidney No	Age	Sex	Etiology
CKD1	39	M	Hypertension, End Stage Renal Disease (ESRD), Diabetes
CKD2	48	F	Hypertension, ESRD, Cardiomegaly, Cerebrovascular accident
CKD3	67	M	Diabetes, Hypertension
NK1	66	F	Cerebrovascular stroke
NK2	75	F	Cerebrovascular stroke
NK3	17	F	Accident

### Cell Growth

Cell growth pattern between NK and CKD cells was compared as described [27]. Briefly, the isolated primary renal cells were plated in 6-well plates with growth medium at a density of 5 x 10^4^ cells per well. After reaching 70–75% confluence, the cells were trypsinized and counted. The cell number was determined at each passage during the 43 days of culture. Triplicate wells were used for cells from each kidney and the assay was repeated on three different kidneys.

### Histology and Immunostaining

Tissues were fixed in 10% paraformaldehyde solution and processed for paraffin embedding. Tissue sections (5μm) were obtained for staining with Hematoxylin and Eosin (H&E), Masson’s trichrome and Periodic Acid-Schiff (PAS). For immunohistochemical analyses, rabbit anti-SOD1 antibody (Santa Cruz Biotechnology, CA, USA) was used at 1:500 dilution using an antibody diluent (Dako North America, Inc., Carpinteria, CA, USA). After incubation with the primary antibody, a biotin-conjugated anti-mouse secondary antibody (Vector laboratories, Burlingame, CA, USA), was applied to the tissue sections and incubated at room temperature for 30 minutes. Tissue sections were then incubated with HRP-conjugated streptavidin (Vector Laboratories, Burlingame, CA, USA) for 30 minutes at room temperature and signals were visualized using the AEC Peroxidase substrate kit (Vector Laboratories, Burlingame, CA, USA).

### Proximal Tubular, Distal Tubular and Podocyte Cell Isolation

To confirm renal specific phenotypes, immunofluorescent staining was performed on the cultured cells using several renal cell markers including aquaporin 1 and podocin, which are expressed by different tubular and podocyte cell population [28, 29]. Proximal tubular cells (PTC), distal tubular cells (DTC) and podocytes were isolated from the heterogeneous cell populations of human primary kidney cells using flow cytometry, (BD FACS Calibur, San Jose, CA, USA). Mouse anti-human Aquaporin 1 (Abcam, Cambridge MA, USA) antibody was used for proximal tubular cells, Tamm-Horsfall protein (THP) (Santa Cruz Biotechnology, CA, USA) was used for distal tubular cells. For podocyte cell isolation, three different markers were used, which included podocyte and endothelial cell marker (PDX) (R&D systems, MN, USA), Wilms’ tumor-1 (WT-1) (Santa Cruz Biotechnology, CA, USA) and nephrin (R&D systems, MN, USA). Primary renal cells were seeded at low density and grown for 3 days in complete media on coverslips. Coverslips were fixed in 4% paraformaldehyde for 10 minutes at room temperature and then washed twice with 1x PBS. Following washes, samples were permeabilized for 15 minutes in 0.25% Triton X-100, then washed with PBS. Slides were incubated for one hour at room temperature with primary antibody at the dilutions (1:300). Samples were washed with PBS and incubated with secondary antibody (1:400 for 1 hour). Cells were then assessed by fluorescence microscopy (Leica, DM 400B, Wetzlar, Germany), digital images (Pro Express 6.3software) were taken. Three random fields were selected per slide and a total of 100 cells were counted in each of the fields. The respective number of proximal and distal tubular cells and podocyte cells were identified based on specific staining and the data has been represented as percentage of positive cells with respective of positive cells counted.

### Scanning Electron Microscopy (SEM)

For surface analysis of renal proximal tubular cells (PTC), primary PTC cells grown as monolayer cultures were washed with PBS, fixed in 2.5% glutaraldehyde in 0.1M sodium cacodylate (pH 7.4) for 2 hours, and stored overnight in 0.1M sodium cacodylate buffer (pH 7.4) at 4°C [30]. Cells were then dehydrated in a series of ethyl alcohol gradients), followed by gold sputtering, and examined using a Hitachi S-4500 Scanning electron microscope (Hitachi Medical Systems America Inc., Twinsburg, OH, USA), magnification x 4780.

### Transmission Electron microscopy (TEM)

In order to assess the ultrastructural properties of renal proximal tubular cells (PTC), standard TEM was performed [31]. Briefly, the kidney tissue was fixed in glutaraldehyde and dehydrated into a series of ethanol and fixed in epoxy resin. The sections were stained with uranyl acetate and analyzed with a Philips TEM400, (Philips Eindhoven, Amsterdam, The Netherlands) transmission electron microscope.

### Oxidative Stress: Glutathione (GSH) Assay by Monochlorobimane

To detect oxidative stress among the NK and CKD cells, GSH assay was performed [27]. Primary renal cells were trypsinized and an equal number of cells was stained with 20μM monochlorobimane (Invitrogen, Carlsbad, CA, USA) in PBS at 37°C for 30 minutes. The cells were then washed with PBS and re-suspended in PBS before loading into a black 96-well plate for fluorescence reading using a spectra-Max MS Microplate Reader (Molecular Devices, Sunnyvale, CA, USA) at excitation and emission wavelength of 355 nm and 460 nm respectively. Triplicate wells were used for cells from each kidney.

### Cellular Sodium Uptake Studies

Sodium uptake function was assessed by the specific proximal tubule receptors as described [32].To determine the levels of sodium transport in human proximal tubular (PTC) cells, confluent monolayers of PTC cells were first pre-incubated with 5 x 10^5^ M ouabain to inhibit Na^+^/K^+^ ATPase for 1 hour prior to sodium uptake studies. The cell monolayer was incubated at 37°C for 30 minutes in a loading medium (culture medium with 5 x 10^5^ M sodium green). At the end of the incubation period, the monolayers were gently washed three times with PBS. A fluorescence spectrum (excitation at 485 nm, emission at 535 nm) of each well was measured using a multi-well plate reader. The fluorescence signal in the system was initially calibrated by exposing the cells to different concentrations of Na^+^ (between 0 to 60 mmol/L) to determine whether the amount of sodium green uptake into the cell reflects the cellular Na^+^ concentration [33]

### Albumin Uptake Assay

Primary renal cell function was assessed by the activation and inhibition of albumin endocytosis mediated by specific proximal tubules receptors. Cell cultures were grown to confluence, and 18–24 hours before performing the assay, the growth medium was replaced with the assay buffer (serum-free, phenol red-free, low glucose (1 g/L) DME medium containing 2 mM glutamine, 10mM HEPES buffer). On the day of assay, the cells were washed twice with the assay buffer consisting of 1.8mM CaCl_2_ and 1mM MgCl_2_ and incubated with the assay buffer for 30 minutes in a humidified chamber at 37°C with 5% CO_2_ to ensure adequate exposure to cofactors. Cells were then exposed to a final concentration of 10–30 μg/mL of rhodamine-conjugated human albumin (ALB-RHO) at 37°C with 5% CO_2_. Wells were washed with ice-cold PBS to stop endocytosis and fixed immediately with 2% paraformaldehyde containing 10 μg/mL Hoechst nuclear dye. The addition of 1μM receptor-associated protein (a specific inhibitor) of albumin uptake in control cultures) served to demonstrate the specificity of the reaction. Induction of ALB-RHO endocytosis was examined by pretreatment of cell cultures with 10^−8^ M human angiotensin II for 4 hours before exposure of ALB-RHO. Cells were then observed by fluorescence microscopy and digital images were acquired for further analysis.

### Statistical Analyses

Statistical analyses were performed using GraphPad Prism (version 6.01). Results are presented as the mean ± S.E.M. Comparisons between the means in different groups were performed using Student *t-* test. Differences were considered to be statistically significant when *p*<0.05.

## Results

### Histopathology of NK and kidneys affected by CKD

**Fig 1:**
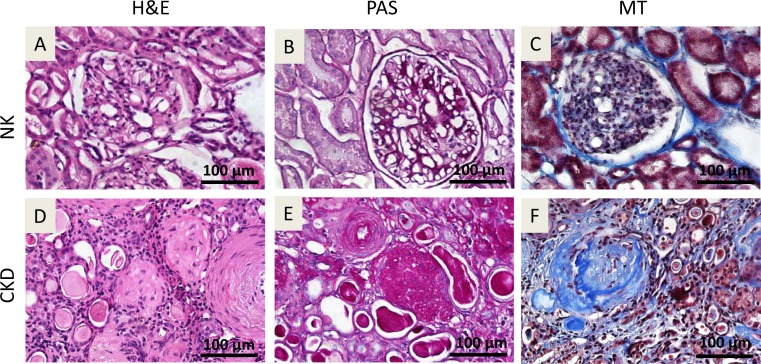
Histology of donor human kidney tissue derived from normal kidneys (NK) and chronic kidney disease-affected kidneys (CKD). H&E staining,(A & D); Periodic acid-Schiff (PAS) staining, (B & E); Masson’s-Trichrome staining (C & F). No major histological abnormalities were seen in the NK tissues. H&E staining of the CKD kidneys revealed a fibrotic cortex with sclerotic glomeruli and scattered chronic infiltration of inflammatory cells. The arterial walls appeared thickened, and the tubules were dilated and filled with pink casts, illustrating renal thyroidization (D). The PAS staining of CKD tissues showed sclerotic glomeruli with collagen deposition and interstitial fibrosis (E). Tubular atrophy and hyalinosis were also observed (D-E). MT staining of the CKD tissues showed collagen deposition in glomeruli (glomerulosclerosis) and interstitium, as well as a thickening of arterial walls (F). Original magnification x40.

Donor characteristics is given in (Table 1). Histopathological analyses of human NK and CKD kidneys (Fig 1) were performed to determine the feasibility of isolating autologous renal cells for transplantation. Hematoxylin & eosin (H&E) stained tissue sections from NK showed normal glomerular tufts with mesangial areas with normal tubules and vascular structure (Fig 1A). Periodic acid-Schiff (PAS) staining (Fig 1B) and Masson’s Trichrome (MT) staining (Fig 1C) of NK sections revealed a fine interstitium in the renal cortex and a thin basement membrane in the glomerulus. H&E staining of the CKD kidneys revealed a fibrotic cortex with sclerotic glomeruli and scattered chronic infiltration of inflammatory cells. The arterial walls appeared thickened, and the tubules were dilated and filled with pink casts, illustrating renal thyroidization (Fig 1D). The PAS staining of CKD tissues showed sclerotic glomeruli with collagen deposition and interstitial fibrosis (Fig 1E). Tubular atrophy and hyalinosis were also observed (Fig 1D and 1E). MT staining of the CKD tissues showed collagen deposition in glomeruli (glomerulosclerosis) and interstitium, as well as a thickening of arterial walls (Fig 1F).

### Morphology and proliferation of cells isolated from NK and CKD kidneys

**Fig 2:**
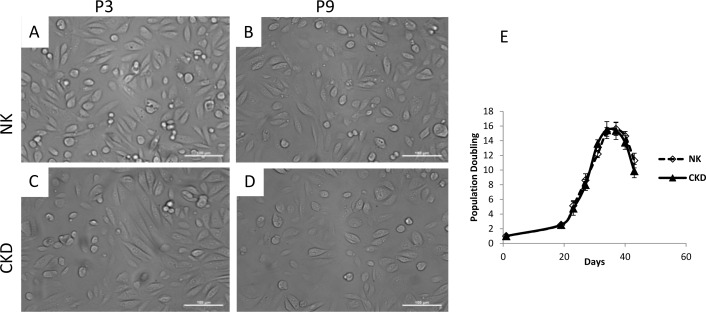
Photomicrograph of primary renal cell cultures derived from NK and CKD kidney at passage 3 (P3) and passage 9 (P9) (A-D). There were no differences in gross cell morphology between NK and CKD kidney cells at passages three (P3) and nine (P9). Original magnification x20; Cell growth curves of NK and CKD kidney derived primary renal cells. Cell growth curve of human NK and CKD cells (2E) from different age donors were counted after achieving confluency, had the same behavior in culture.

Renal cells from each group (n = 3) were isolated, cultured, and expanded for 43 days. Cell morphology was assessed microscopically at each passage, and a cell proliferation assay was performed throughout the cell expansion process. Cells cultured from NK and CKD kidneys showed similar phenotypes and proliferation kinetics. There were no significant differences in gross cell morphology between NK and CKD kidney cells at passages three (P3) and nine (P9) (Fig 2A to 2D). Furthermore, cells obtained from different donors (Table 1) did not show any differences in their growth patterns (Fig 2E). The cells were cultivated for an average of 43 days and the population maximum doubling in this period was 37 days for both NK and CKD derived primary cells. The population doubling during this period was 15.6 ± 0.87 and 15.35 ± 1.18 times that of NK-derived cells and CKD kidney-derived cells respectively. There was no significant difference (P≤0.58 and P≤0.60) between the growth characteristics of cells derived from NK and CKD. Additionally, the *in vitro* growth of both NK- and CKD-derived cells decreased after 37 days.

### Renal cell characterization of NK and CKD using various cellular markers

**Fig 3:**
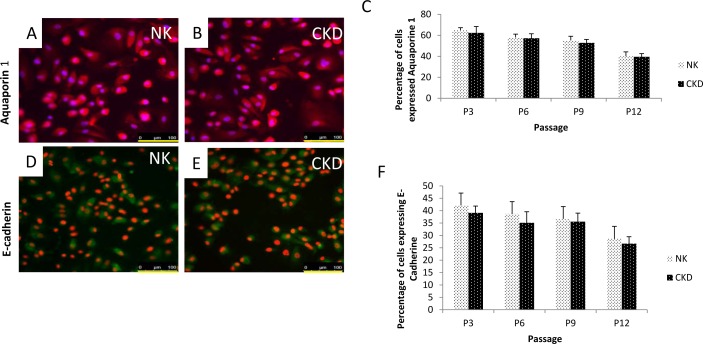
Characterization of isolated primary renal cells from NK and CKD kidneys using cell-specific markers. Florescent antibody staining was carried out on passage 3 (P3) cells. Staining with proximal tubular marker Aquaporin-1 (A-B); Quantitation of proximal tubular cells (Aquaporin-1) among the total isolated primary renal cells at different passages from P3 to P12; (C) Distal tubular marker E-cadherin1 staining of primary renal cells from NK and CKD kidneys. N = 3; (D-E) Quantitation of distal tubular cell (E-cadherin) among the total isolated primary renal cells at different passages from P3 to P12 (F). The overall amounts (percentage) of proximal tubular cells and distal tubular cell were similar in the renal cell population derived from NK and CKD kidneys. Original magnification x20.

To characterize the heterogeneous population of primary renal cells, we used several specific markers. Aquaporin1 and E-cadherin1 were used to identify proximal tubular cells and distal tubular cells (Fig 3A and 3B) in NK and CKD samples, respectively. Based on aquaporin 1 expression, we observed that the quantity of proximal tubular cells ranged from 65 ± 2.2% at P3, to 41.2 ± 4.1% at P12 in the NK-derived cells and 62.3 ± 6.2% at P3 to 39.5 ± 3% at P12 in the CKD-derived cells. There was no significant difference (P≤0.18) between NK and CKD cells at any of the passages that were analyzed (Fig 3C). Comparatively, using E-cadherin 1 expression as a distal tubular cell marker, we observed that the quantity of this cell type in NK-derived cultures ranged from 41.4 ± 3% at P3 to 28.2 ± 1.8% at P12 (Fig 3D and 3E), while in CKD-derived cells we observed a range from 37.3 ± 2.8% at P3 and 26.1 ± 1.5% at P12. There was no significant difference (P≤0.42) between the NK and CKD cells at any of the passages analyzed. Therefore, the number of proximal and distal tubular cells appeared to decrease with increasing passages, P3 to P12, (Fig 3F) in both NK and CKD tissues. The relative number of proximal and distal tubular cells, however, remained the same irrespective of the pathophysiological condition of the kidney. This is an indication that healthy, functional autologous renal tubular cells can be isolated and cultured from diseased kidneys.

**Fig 4:**
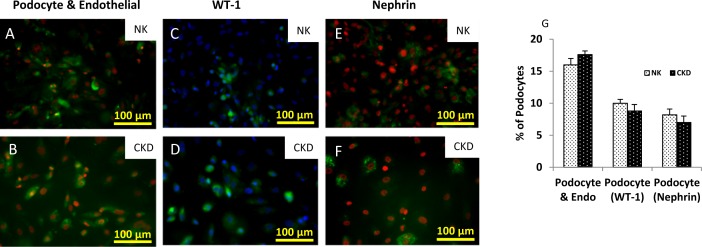
Identification of Podocytes among the primary renal cells from NK and CKD using antibody-based staining of cell surface markers. (A-B) Podocytes and endothelial cells identified by Podocalyxin (PDX) staining; (C-D) Podocytes stained using Wilms’ tumor (WT-1) antibody; (E-F) Staining of Podocytes using another cell-specific marker Nephrin; Quantitation of Podocytes among the primary renal cells isolated from NK and CKD kidneys at P3. Note that use of WT-1 and Nephrin antibodies resulted in slightly different levels of Podocytes detection amount the renal cell population (G). However, the relative amounts of Podocytes in both NK and CKD kidney derived cells were similar.

For podocyte characterizations (Fig 4), three different markers were used which included a podocyte and endothelial cell marker (PDX) [26], Wilms’ tumor-1 (WT-1), and nephrin (Fig 4A to 4F). The expression levels of podocytes markers were analyzed by fluorescent microscopy using different successive passages. Podocytes and endothelial cells in both tissue types (NK and CKD) expressed the PDX marker [26]. PDX staining and quantification of cells at P3 showed that 16 ± 1% of the cell population isolated from NK were either podocytes or endothelial cells, while the number of podocytes or endothelial cells isolated from CKD was 17.6 ± 0.57%. This result indicated that no significant difference (P ≤ 0.06) was observed between the two groups (Fig 4G). WT-1 and nephrin are known as cell-surface markers for podocytes, [34] and they were used for selective detection and quantification of podocytes in P3 cells. Staining for WT-1 in podocytes from NK and CKD populations, revealed a respective podocyte presence of 10 ± 1 and 8.23 ± 0.6%, which again demonstrated no significant difference (P ≤ 0.06) between the groups (Fig 4G). Finally, nephrin staining of NK-derived cells showed that 8.2 ± 0.9% of the NK-derived cells and 7 ± 1% of cells from CKD-derived cells were confirmed as podocytes. This result highlights that there is no significant difference between the groups P ≤0.06, (Fig 4G).

**Fig 5:**
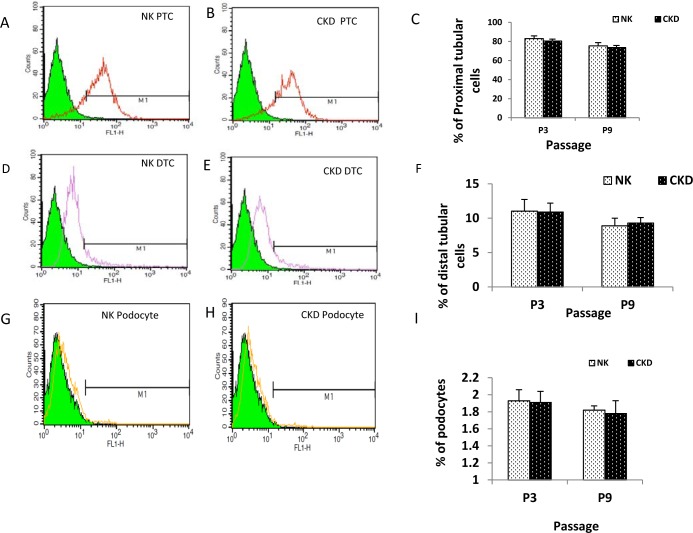
Characterization of specific renal cells among the total renal cells isolated from NK and CKD kidneys. Cell-type specific antibodies and FACS was used to isolate proximal tubular cells (A-B), distal tubular cells (D-E) and podocytes (G-H) in different passages (P3 and P9) of cultured renal cells from NK and CKD kidneys. FACS-based quantification percentage of proximal tubular cells in passage 3 and 9 cells (C), percentage of distal tubular cells (F), percentage of podocytes (I). The result highlights that there is no significant difference between the groups.

**Fig 6:**
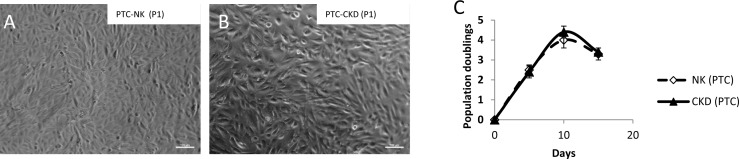
Photomicrograph of proximal tubular cells (PTC) purified from primary cell cultures that were originally derived from NK kidneys (A) and CKD kidneys (B) at passage 1 (P1) original magnification x10. Consolidated growth curve of proximal tubular cells isolated from NK and CKD human renal cells (C). Proximal tubular cells from different age donors were counted after achieving confluency, had the same behavior in culture.

We utilized fluorescence-activated cell sorting (FACS), with cell type-specific antibodies conjugated to fluorescein isothiocyanate (FITC) (Fig 5A to 5C), to isolate individual cell populations for further studies. Sorting of NK-derived primary renal cells resulted in the isolation of 83.1 ± 2.8% proximal tubular cells from P3 and 75.75 ± 0.9% proximal tubular cells from P9. The number of proximal tubular cells isolated by FACS from CKD-derived primary renal cells was 80.3 ± 4.6% for P3 (P ≤0.54) and 73.7 ± 0.7% for P9 (P ≤0.12) respectively. Similarly, sorting of NK-derived distal tubular cells showed that 11.03 ± 1.7% of P3 cells and 8.9 ± 1.3% of P9 cells were identified as distal tubular cells (Fig 5D to 5F), and sorting of CKD-derived distal tubular cells showed that 10.9 ±1.1% of P3 cells and 9.3 ± 0.8% of P9, P ≤0.93 and P ≤0.67. FACS was also used to successfully isolate the population of podocytes from both NK- and CKD-derived primary renal cells at P3 and P9 (Fig 5G to 5I). The percentage of podocytes contained in the NK- and CKD-derived cells at passage 3 was found to be 1.93 ± 0.13 and 1.91± 0.13 (P ≤0.90), respectively. Interestingly, the percentage of podocytes at passage 9 was also similar; 1.82±0.05 and 1.78±0.15% (P ≤0.70), for NK- and CKD-derived cells, respectively. Overall, the number of proximal tubule cells, distal tubule cells, and podocytes that were isolated using FACS was similar between the NK- and CKD-derived primary renal cells. This result supports our earlier observation that it is possible to isolate specific renal cells for cell-based therapy applications from diseased (CKD) kidneys. The proximal tubular cells isolated from different donors and kidney types (NK and CKD kidneys) also showed similar phenotypes (Fig 6A and 6B). The proliferation kinetics, growth curves, and doubling times were similar for proximal tubular cells (PTC) obtained from NK and CKD kidneys (Fig 6C). The population doubling time of the PTC from NK was 4 ±0.4, and for the CKD was 4.4±0.3 (P ≤0.76).

### Ultra structural evaluation of isolated proximal tubular cells

**Fig 7:**
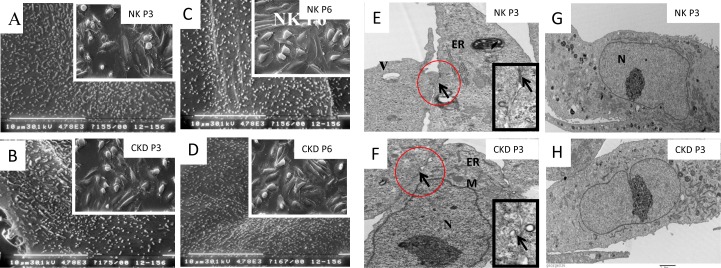
Ultrastructural analysis of renal proximal tubular cells using Scanning Electron Microscopy (SEM) and Transmission Electron Microscopy (TEM). SEM analysis showing long microvilli on the apical surface membrane of a Proximal tubular cells derived from a NK kidney at passage 3 (A) and passage 6 (C) and the same cell type from a CKD kidney at passage 3 (B) and passage 6 (D); Upper panel shows the cellular morphology magnified x630. TEM analysis showing the integrity of tight junction (arrow) in proximal tubular cells isolated from primary renal NK kidneys (E) and CKD kidney cells (F) at passage 3 (P3) magnified x4780; TEM micrograph showing the ultrastructure of nucleus “N” and other intracellular components in proximal tubular cells of NK (G) and CKD (H) kidneys are similar morphology, magnified x11000.

Scanning electronic microscopy (SEM) analysis of the cultured primary human proximal tubular cells demonstrated microvilli on the apical surface, constituting a brush border indicating cellular polarity; thus, the microvilli can act as a mechano-sensor for fluid flow. The isolated proximal tubule cells from the NK and CKD kidneys possessed long microvilli structures on the apical surface, both at P3 and P9 (Fig 7A to 7D). No morphological differences were observed between the microvilli isolated from the NK- and CKD-derived cells. Similarly, cell junction analysis using transmission electron micrograph (TEM) showed an identical appearance of tight junctions (TJ) and desmosomes (D), (Fig 7E and 7F) in the proximal tubule cells derived from both NK (P3) and CKD (P3) kidneys.

Additionally, TEM analysis showed that the nuclei (N) and other intracellular organelles in the NK- and CKD-derived proximal tubule cells are similar (Fig 7G and 7H). Hence, the ultra-structural analysis supports our hypothesis that proximal tubular cells derived from a diseased kidney (CKD) show potential for therapeutic use.

### Oxidative Stress in NK and CKD tissues and cells

**Fig 8:**
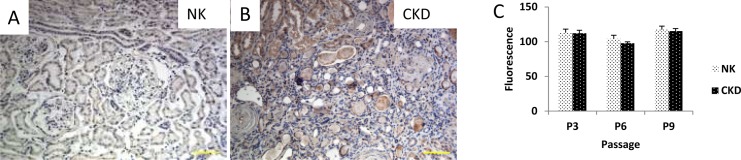
Oxidative Stress in NK and CKD kidney derived tissues. Immunohistochemical staining to detect Superoxide dismutase 1 (SOD1) expression in human renal tissues from NK (A) and CKD (B) kidneys. Quantitation of oxidative stress in primary renal cells derived from NK and CKD kidneys using a Glutathione (GSH) assay that utilizes a fluorescent dye Monochlorobimane (MCB), (C). GSH was assayed in cells of passage 3 (P3) to (P9) and 12. The Glutathione levels in renal cells derived from both NK and CKD kidneys were almost similar during the entire cell culture

Superoxide dismutase (SOD) is an anti-oxidative enzyme normally present in living cells [35]. Antioxidant mechanisms that can be either enzymatic including catalases, dismutases and peroxidases or non-enzymatic such as vitamin A, C or E are critical to protecting cells against ROS-induced damages [36]. However, the mechanism(s) of SOD’s renal cellular action in response to oxidative stress, in both NK and CKD tissues, is unknown. The expression of superoxide dismutase-1 (SOD1) was examined in the NK and CKD kidney tissues, and as expected, the CKD tissue expressed higher SOD1 compared to the NK tissues (Fig 8A and 8B). Glutathione (GSH) is an important intracellular antioxidant that protects against a variety of different antioxidant species [37]. Oxidative stress was also assessed in the primary human renal cells isolated from the NK and CKD kidney tissues using the fluorescent dye, monochlorobimane (MCB), which can be detected when bound to GSH. In this study the expressions of GSH were at similar levels in both NK- and CKD-derived renal cells at P3 to P9. The GSH levels in the NK cells, at P3 and P9, were 113±5.5 and 118.4±1.9 respectively as measured by fluorescence. Similarly, GSH levels in the CKD cells at P3 and P9 were 112.2±4.3 (P ≤ 0.74) and 106.6±2.2 (P ≤ 0.32), respectively (Fig 8C).

### Sodium uptake and protein transport

**Fig 9:**
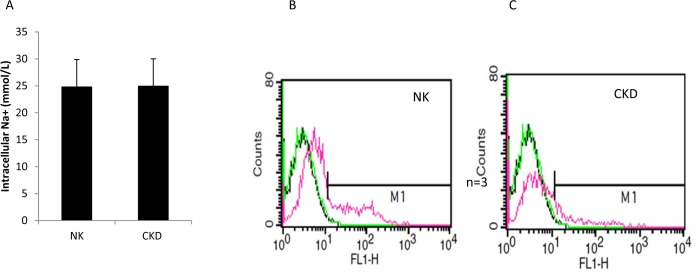
Quantitative uptake analysis of primary proximal tubular cells (PTC) derived from NK and CKD kidneys confirms specific uptake of Na^+^ by the cells with no significant difference. Ouabain treatment increases sodium uptake by inhibiting Na/K ATPase. FACS analysis of Intracellular Na^+^ uptake using the cell permeant Sodium Green Tetra-acetate in (PTC) cells from NK and CKD kidneys (B-C) were similar.

**Fig 10:**
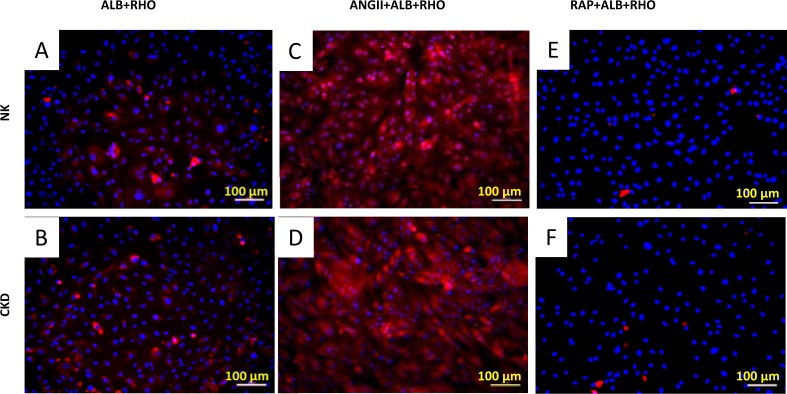
Measurement of protein transport in primary renal cells from NK (A) and CKD (B) kidneys, as measured by uptake of rhodamine-conjugated albumin (ALB-RHO).Enhancement of ALB-RHO in the presence of Angiotensin II (ANGII) in the cells derived from NK (C) and CKD (D) kidneys. Reduction of ALB-RHO uptake by the NK (E) and CKD (F) kidneys derived cells in the presence of receptor-associated protein (RAP). NK and CKD kidneys derived cells Showing the same behavior of protein transport in culture.

To further determine the functional similarities or differences between the primary renal cells from NK and CKD, intracellular sodium (Na^+^) uptake assays were performed. Fluorescent microscopy revealed that the majority of the expanded human renal cells exhibited reabsorptive capacities via the uptake of sodium green. Primary human renal proximal tubular (RPT) cells generate and regulate cellular Na^+^ absorption. Treatment with ouabain increased the intracellular Na+ ions concentration [33]. This result supports the quantitative studies that illustrated significant levels of sodium uptake by renal cells [28]. The addition of ouabain resulted in a comparable increase in intracellular Na^+^ uptake in both NK and CKD derived cells, 24.4 ± 2.26 mmol/L and 25±1.95 mmol/L, respectively, showing no significant difference (P ≤0.87), (Fig 9A). These results indicate that no significant functional difference was observed between the NK and CKD derived renal cells. FACS analysis of Na^+^ uptake by these cells was further assessed using sodium green (Invitrogen, CA, USA), which is a visible light-excitable Na^+^ indicator. The results showed a similar Na^+^ uptake pattern for both the NK and CKD derived renal cells (Fig 9B and 9C). Renal proximal tubular cell function was assessed by the activation and inhibition of albumin endocytosis, mediated by specific proximal tubule receptors, megalin and cubilin [32]. In addition, protein transport by the NK and CKD derived renal cells was analyzed using rhodamine-conjugated albumin (ALB-RHO assay) [38]. The NK and CKD derived renal cells showed similar patterns of rhodamine-conjugated albumin uptake (Fig 10A and 10B). The specificity of the reaction was confirmed by the enhanced uptake of ALB-RHO, with angiotensin II (ANGII) (Fig 10C and 10D), and by the blockage of ALB-RHO uptake in the presence of receptor associated protein (RAP), (Fig 10E and 10F).

## Discussion

This study is based on the concept that autologous cell-based approaches can improve and restore renal function [8, 9, 38–40]. Studies in both the liver and kidney have indicated that resident parenchymal cells are more efficient at restoring function to damaged tissue after injury than the ectopically-derived stem and progenitor cells [36–38]. Several initial cell-therapy studies using renal cells isolated from normal kidney (NK) showed functional improvement. Previously, the ability of autologous renal cells to improve kidney function in a rodent renal failure model has been demonstrated [8]. We further propose that healthy cells derived from diseased kidneys would be better suited for an autologous cell-based therapy approach to treat patients with CKD or ESRD. To date, there has been no sufficient evidence to show that primary renal cells derived from CKD kidneys are functionally similar to cells derived from a normal healthy kidney (NK). Use of primary renal cells from a diseased kidney could prevent morbidity associated with use of allogeneic kidneys as cell sources, enable more autologous cell-based therapy, and reduce dependence on organ transplants for treating CKD or ESRD conditions.

In this study, we demonstrated that human renal cells isolated from the NK and CKD tissues showed no differences in phenotypic and functional characteristics. Cells from both sources displayed similar morphologies, proliferation kinetics, and expression of kidney specific markers. A majority of the isolated primary renal cells was proximal tubular cells with very few glomerular cell types present. Complexity and heterogeneity are difficult variables to replicate in the study of renal function; however, we have been able to characterize several of the cells types using specific antibodies in the heterogeneous mixture of NK and CKD derived cells. The majority of cells in the isolated renal cell population were proximal tubular and distal tubular cells. The proximal tubular cells constituted a phenotypically distinct, scattered cell population that participates in tubular regeneration [41]. The proximal tubular cells contain aquaporin 1, membrane-inserted water channel protein, which plays an important role in the reabsorption of water from the renal tubular fluid. The renal proximal tubules are responsible for the reabsorption of water, Na^+^, glucose, amino acids, and proteins from the glomerular filtrate [42, 43]. The podocyte is the most differentiated cell type in the glomerulus, which forms a crucial component of the glomerular filtration barrier [44]. However, in the current study no significant difference was observed in the percentage of podocytes formed from the primary renal cells obtained from both (NK and CKD) types of kidneys.

Another observation from the present study was that cellular oxidative stress in the CKD tissues was related to the antioxidant enzyme, superoxide dismutase (SOD1). The superoxide dismutase family of antioxidant enzymes is a major defense system against the superoxide anion, converting superoxide into hydrogen peroxide (H_2_O_2_) and molecular oxygen (O_2_) [45]. The results in this study demonstrated no significant cellular stress in both NK and CKD derived primary renal cells and similar effects of glutamate and malate dehydrogenase were observed, as assessed by fluorometric measurement of monochlorobiamine (MCB) sulphate procedure [27, 46]. Functional measurements of NK and CKD derived primary cells showed similar Na^+^ uptake in the presence of the Na^+^/K^+^ ATPase inhibitor ouabain [28, 33].

The serum proteins absorbed from the filtrate are composed mostly of albumin [43–44]. Albuminuria is an indicator and pathogenic factor in CKD progression. The cells isolated from the NK and CKD kidneys showed similar protein transport functions, as analyzed from the uptake of rhodamine-conjugated albumin in the presence of angiotensin II. The glycoproteins, cubulin and megalin, constitute important endocytic receptors localized to the kidney proximal tubule to enhance the uptake of albumin [38]. Our results show that the proteins associated with uptake of Na^+^ and albumin in the proximal tubular region of the CKD-derived cells are functionally similar to NK-derived cells. Likewise, the phenotypic characteristics of the tight junctions were similar between NK- and CKD-derived renal cells. The proteins present in the tight junction include claudin and occludin, which enable the barrier function and permit selective paracellular transport. Through injury, multiple activated signaling pathways are activated, which lead to the phosphorylation of tight junction proteins and disruption of the complex. Maintenance of a polarized phenotype and transport functions are required for healthy epithelial function, but both are altered in numerous disease processes [47, 48]. Our results showed that the tight junctions in the NK- and CKD-derived renal cells are similar in structure, and there are no cellular changes observed in polarity and the microvilli patterns. Additionally, the cell surface and the integrity of the cellular components are maintained in both NK and CKD derived primary renal cells. While we observed no significant difference between the isolated NK and CKD cells in the appearance or function, the number of kidneys used in this study was low, and the etiology of CKD varies from patient to patient. Further studies using larger samples of CKD patients are necessary to accurately determine the utility of this approach.

In conclusion, human renal cells isolated from normal and diseased kidney tissues showed similar phenotypic and functional characteristics. We have successfully demonstrated that renal cells isolated from diseased kidneys can be expanded *in vitro* without incurring changes to their cellular properties when compared to normal renal cells. This study supports the possibility of using autologous renal cells isolated from diseased kidneys as a reliable cell source for the treatment of renal failure, including CKD and ESRD. Further studies are warranted to look at the regenerative potential of these cells in preclinical models to assess safety and efficacy before clinical application in human CKD patients can be established.
